# Hypothyroidism and the risk of breast cancer recurrence and all-cause mortality - a Danish population-based study

**DOI:** 10.1186/s13058-019-1122-3

**Published:** 2019-03-22

**Authors:** Anne Mette Falstie-Jensen, Anders Kjærsgaard, Ebbe Laugaard Lorenzen, Jeanette Dupont Jensen, Kristin Valborg Reinertsen, Olaf M. Dekkers, Marianne Ewertz, Deirdre P. Cronin-Fenton

**Affiliations:** 1 Department of Clinical Epidemiology, Aarhus University Hospital, Olof Palmes Allé 43-45, DK-8200 Aarhus N, Denmark; 2 Department of Oncology, Odense University Hospital, Odense, Denmark; 3 Institute of Clinical Research, University of Southern Denmark, Odense, Denmark; 4 National Advisory Unit on Late Effects after Cancer Treatment, Department of Oncology, Oslo University Hospital, Norwegian Radium Hospital, Oslo, Norway; 5 Department of Epidemiology, Leiden University Medical Center, Leiden, The Netherlands

**Keywords:** Breast cancer, Breast cancer recurrence, Breast cancer survival, Epidemiology

## Abstract

**Background:**

Hypothyroidism may occur as a late effect of breast cancer-directed treatment, particularly after radiotherapy, but little is known whether hypothyroidism affects the prognosis after breast cancer. We investigated the association between hypothyroidism and breast cancer recurrence, and all-cause mortality.

**Methods:**

In this population-based cohort study, we used national medical registries to identify all Danish women 35 years or older diagnosed with stage I–III, operable breast cancer between 1996 and 2009. Hypothyroidism was defined as hospital diagnoses ascertained via diagnostic codes, or as prescriptions for levothyroxine. Two analytic models were used: (i) hypothyroidism present at the time of the breast cancer diagnosis (prevalent) and (ii) hypothyroidism diagnosed during follow-up as a time-varying exposure lagged by 1 year (incident). Breast cancer recurrence was defined as any local, regional, or distant recurrence or contralateral breast cancer. All-cause mortality included death from any cause in any setting. We used Cox regression models accounting for competing risks to compute adjusted hazard ratios (HRs) and 95% confidence intervals (CIs) of breast cancer recurrence and all-cause mortality.

**Results:**

The study cohort included 35,463 women with breast cancer with 212,641 person-years of follow-up. At diagnosis, 1272 women had hypothyroidism and 859 women developed hypothyroidism during follow-up. In total, 5810 patients developed recurrent breast cancer. Neither prevalent nor incident hypothyroidism was associated with breast cancer recurrence (adjusted HR_prevalent_ 1.01, 95% CI 0.87–1.19; adjusted HR_incident_ 0.93, 95% CI 0.75–1.16, respectively). Furthermore, no differences were seen for all-cause mortality for prevalent or incident hypothyroidism (adjusted HR_prevalent_ 1.02, 95% CI 0.92–1.14, and HR_incident_ 1.08, 95% CI 0.95–1.23, respectively). Stratification by menopausal status, oestrogen receptor status, chemotherapy, or radiotherapy did not alter the estimates.

**Conclusions:**

Hypothyroidism present at diagnosis or during follow-up was not associated with breast cancer recurrence or all-cause mortality in women with breast cancer. Our findings provide reassurance to patients and their physicians that hypothyroidism is unlikely to impact on the clinical course of breast cancer or survival.

**Electronic supplementary material:**

The online version of this article (10.1186/s13058-019-1122-3) contains supplementary material, which is available to authorized users.

## Background

Breast cancer is one of the most common malignancies in women, worldwide. Over the last 25 years, mortality has decreased by 36% leading to an increased number of breast cancer survivors [1]. This considerable decline is likely attributable to advances in mammographic screening and improved surgical, radiation, and adjuvant therapies [2]. However, cancer and cancer-directed treatment can incur serious long-term negative health effects. Thus, it is critical to monitor the potential impact of such late effects on breast cancer prognosis.

Hypothyroidism is a common hormone deficiency, characterised by insufficient production of triiodothyronine and thyroxine [3]. The diagnosis of hypothyroidism is confirmed with blood tests measuring thyroid-stimulating hormone and thyroxine levels. Hypothyroidism requires substitution therapy. Despite adequate biochemical control, symptoms like fatigue or disturbed concentration do not always resolve. Hypothyroidism is diagnosed in about 3% of the population, more frequently in women, and risk increases with age; thus, some breast cancer patients develop hypothyroidism long before their breast cancer is diagnosed [4].

Hypothyroidism is a well-documented late effect after radiation therapy in head and neck cancer [5]. Consequently, a link between breast cancer treatment and subsequent risk of hypothyroidism has been discussed, initially by case reports published on breast cancer patients who developed hypothyroidism years after treatment [6–10]. Later, observational studies from Europe and the USA suggested that breast cancer patients may have a higher risk of hypothyroidism during follow-up [11–13]. Furthermore, several studies have linked types of cancer-directed treatments with hypothyroidism [14–18] and radiotherapy, particularly among those receiving radiotherapy to the supraclavicular region [12–14, 19]. However, the scientific literature on the association of hypothyroidism with breast cancer prognosis is sparse. Laboratory-based animal models have shown that induced hypothyroidism without the use of substitution therapy may correlate with smaller, less-invasive tumours [20–22]. Thus, breast cancer patients with hypothyroidism may have a lower risk of breast cancer recurrence.

The aim of this study was first to investigate the association between hypothyroidism prevalent at breast cancer diagnosis, or incident during follow-up, and the subsequent risk of breast cancer recurrence in a large population-based cohort of breast cancer patients. Second, we investigated the association between hypothyroidism and all-cause mortality.

## Methods

This study was approved by the Danish Data Protection Agency (Aarhus University, journal number 2016-051-000001, running number 437), the Danish Medicines Agency, and the Danish Breast Cancer Group (DBCG). According to Danish Law, ethical approval is not necessary because the study uses routinely collected registry data.

In Denmark, a unique civil personal registry number is assigned to all citizens at birth or immigration, enabling accurate and unambiguous individual-level record linkage across all public registries, medical as well as non-medical [23]. Due to tax-funded healthcare, all 5.6 million citizens have free access to public hospitals, which covers more than 95% of hospitalisations including all emergencies.

### Source population

We used the DBCG clinical database to ascertain information on all women 35 years or older with incident stage I-III, operable breast cancer on protocol treatment and diagnosed between January 1, 1996, and December 31, 2009 [24]. The DBCG was established in 1977 to optimise diagnostic and therapeutic procedures across the country and to improve breast cancer prognosis [25]. All patients with invasive breast cancer in Denmark are included prospectively, and registration completeness has increased over the years to reach ~ 95% for the last decade [26, 27]. The treating physicians are responsible for entering pre-specified data on patient, tumour, and treatment characteristics. We excluded women with prevalent hyperthyroidism at the time of the breast cancer diagnosis from the analyses.

### Thyroid disease

We defined hypothyroidism as a diagnostic code of hypothyroidism or the redemption of at least two prescriptions of levothyroxine. Information on diagnostic codes (International Classification of Diseases (ICD) 8: 244.00-244.03, 244.08, and 244.09, and ICD-10: E03.2-E03.9, and E89.0) was obtained from the Danish National Registry of Patients (DNRP) covering information on all discharge diagnoses for inpatient hospital contacts since 1977 and outpatient and emergency room hospital contacts since 1995 [28]. We used the Danish National Prescription Registry (DNPR) to identify patients redeeming at least two prescriptions (Anatomical Therapeutic Classification (ATC) code: H03A) and to include patients treated for hypothyroidism but not necessarily recorded in the DNRP [29].

To exclude women with hyperthyroidism from the study population, we identified diagnoses of hyperthyroidism in the DNRP by ICD-8: 242.01-242.29 and ICD-10: E05-E05.9 and E05.0B, or in the DNPR by redemption of at least two anti-thyroxine prescriptions (ATC codes: H03BB01, H03BB02, and H03BA02) during follow-up.

### Outcomes

We used the DBCG definition of breast cancer recurrence as any local, regional, or distant recurrence or contralateral breast cancer [25, 26]. Regional recurrence includes recurrence in the same site as the first primary breast cancer in the axilla, supraclavicular, or parasternal lymph node region. All other recurrences are regarded as distant. When a new tumour is detected, a biopsy is taken for pathological assessment. The decision whether the new tumour is a recurrence of a previous cancer or a new primary tumour is based on this assessment in accordance with the clinical guidelines. Due to systematic follow-up, all cases of recurrence are reported in the DBCG including the date and anatomical site of recurrence. The systematic follow-up for patients with operable disease includes a clinical evaluation, biannually for the first 5 years and annually up to 10 years after diagnosis.

All-cause mortality included death from any cause in any setting. We obtained data on mortality from the Danish Civil Registration System, which has registered information on vital and migration status on all Danish inhabitants since 1968 [23].

### Covariates

From the DBCG, we retrieved clinical and treatment characteristics: menopausal status at diagnosis (pre/post), histological grade (a composite score including tubule formation, mitoses, and nuclear pleomorphy) (low, moderate, and high), lymph node status (N0, N1–3, N4+), tumour oestrogen receptor (ER) status (positive ≥ 10%/negative 0–9%), HER-2 status (classified according to immunohistochemistry (Hercept test) and by fluorescent in situ hybridisation (FISH) and available from 2007) (positive HER-2 score = 3 and FISH ≤ 2.00, negative HER-2 score ≤ 2 and FISH ≤ 2.00), and chemo-, radio-, and endocrine therapy (ET) (yes, no) (intention-to-treat information). We summarised the type of primary surgery and radiotherapy into a joint variable (mastectomy with radiotherapy, mastectomy without radiotherapy, lumpectomy) and ER and ET as a joint variable (ER+/ET+, ER+/ET−, ER−/ET+, and ER−/ET−).

We ascertained information on comorbidities diagnosed up to 10 years before primary breast cancer diagnosis from the DNRP. A modified comorbidity score was calculated for each patient according to the Charlson Comorbidity Index excluding cancer in the index score [30]. Based on the score, three categories were computed (no comorbidity, low (score of 1 or 2), and high (score ≥ 3)).

### Statistical analyses

**Fig. 1:**
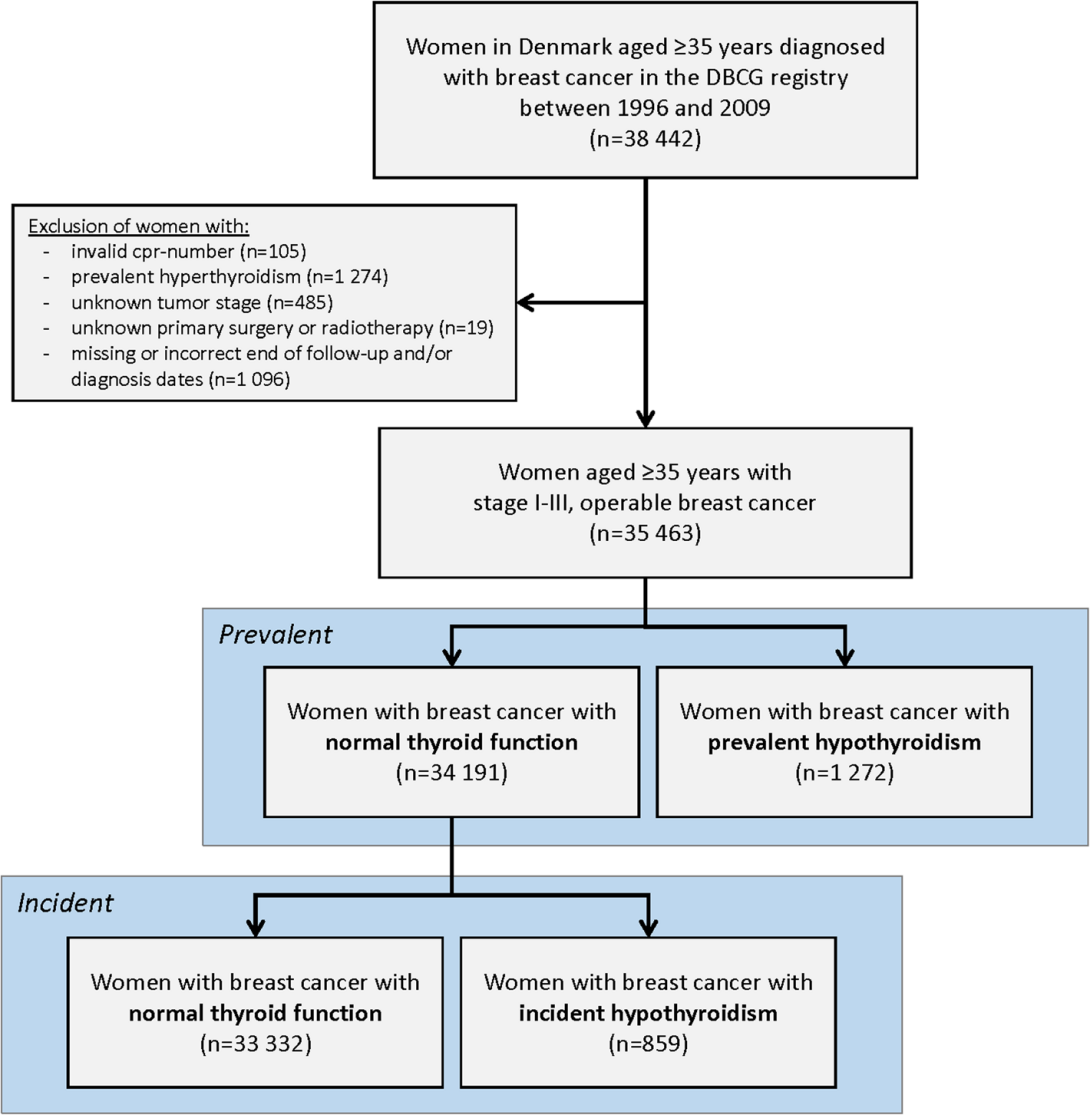
Flowchart of including women for the study of breast cancer recurrence

We used a prevalent and an incident model for hypothyroidism as illustrated in Fig. 1. In the prevalent model, hypothyroidism was included as a baseline exposure. Women with a clinical diagnosis of hypothyroidism and/or redeemed prescriptions for thyroxine before or at the time of the breast cancer diagnosis were considered to have prevalent hypothyroidism. For incident hypothyroidism, exposed person-time started at the date of hypothyroidism or once a patient had redeemed at least two thyroxine prescriptions with prescriptions treated as a time-varying exposure lagged by 1 year (please see Additional file 1) [31].

Person-time at risk of recurrence was computed from the date of primary breast cancer surgery (index date) and continued to the date of breast cancer recurrence, death, emigration, hyperthyroidism, 10 years, or the first of June 2015 whichever came first. For all-cause mortality, person-time at risk was calculated from the index date and continued to the date of death, emigration, hyperthyroidism, 10 years, or the first of June 2015 whichever came first.

Within categories of patient, clinical, and treatment characteristics, we examined the frequency and proportion of breast cancer patients according to thyroid status at baseline and during follow-up using euthyroidism as a reference group.

We used Cox regression models to compute crude and adjusted hazard ratios (HR) including 95% confidence intervals (95% CI) comparing the risk of recurrence and all-cause mortality, respectively, according to thyroid status (normal thyroid versus prevalent/incident hypothyroidism) [32]. The proportional hazard assumption was checked by visual inspection of the log of the estimated survivor function in the models involving prevalent hypothyroidism (Additional file 2). The models accounted for competing risks and included adjustments for potential confounding covariates including age at diagnosis (continuous), menopausal status, UICC stage, ET/ER status, surgery type, receipt of chemotherapy, histologic grade, comorbidity, and use of simvastatin or aspirin, respectively. Simvastatin and aspirin use were modelled as time-varying covariates lagged by 1 year after redemption of a prescription and lasting for 1 year. Simvastatin and aspirin use were included in the adjusted models as they have been linked to breast cancer prognosis, and adherence to one medication may correlate with adherence to another prescription [33, 34]. Due to the low number of events in the incident model, we used a directed acyclic graph (DAG) to identify the relevant confounders for recurrence (please see Additional file 3). The final DAG adjusted model included age at diagnosis, UICC stage, chemotherapy, type of primary surgery, and ER/ET status.

In both models, we investigated potential effect measure modification stratifying by menopausal status, chemotherapy, and ET/ER use and for the incident model also stratifying by radiotherapy. In sensitivity analyses, we increased the lag time from 1 to 2 years. For recurrence, we also performed sensitivity analyses restricting to patients who had hypothyroidism 2, 5, and 10 years before their breast cancer diagnosis to investigate the association of duration of hypothyroidism with breast cancer recurrence.

All statistical analyses were performed using SAS version 9.4 (SAS Institute, Cary, NC).

## Results

In the DBCG registry, 38,442 Danish women ≥ 35 years were diagnosed with non-metastatic breast cancer between 1996 and 2009. Due to reasons outlined in Fig. 1, 2979 (8%) women were excluded, and so the final study population included 35,463 women with early stage I-III, operable breast cancer.

At baseline, 34,191 (96%) women with breast cancer had a normal thyroid and 1272 (4%) had hypothyroidism with a follow-up time of 205,529 and 7112 person-years, respectively. Women with normal thyroid function were followed for a median of 6.0 years, and women with prevalent hypothyroidism for 5.6 years. Women with prevalent hypothyroidism were older, were more frequently post-menopausal, had more comorbidity, were less likely to be assigned chemotherapy, and were more often simvastatin users compared with their euthyroid counterparts (Table 1).

**Table 1:** Baseline characteristics of women diagnosed with stage I–III, operable breast cancer in Denmark from 1996 to 2009, according to thyroid status at the time of breast cancer diagnosis

Characteristics	Prevalent hypothyroidism				Incident hypothyroidism			
	Thyroid status		Follow-up time^1^		Thyroid status		Follow-up time^1^	
	Normal (*n* = 34,191)	Hypothyroidism (*n* = 1272)	Normal	Hypothyroidism	Normal (*n* = 33,332)	Hypothyroidism (*n* = 859)	Normal	Hypothyroidism
Calendar year of diagnosis, *n* (%)								
1996–1999	8059 (24)	199 (16)	47,917	1166	7888 (24)	171 (20)	47,339	679
2000–2004	11,522 (34)	413 (32)	76,332	2562	11,226 (34)	296 (34)	75,204	1127
2005–2009	14,610 (43)	660 (52)	81,280	3383	14,218 (43)	392 (46)	80,004	1276
Age at diagnosis, *n* (%)								
35–40 years	1172 (3)	11 (1)	7366	62	1147 (3)	25 (3)	7277	89
40–49 years	6134 (18)	115 (9)	40,077	708	5961 (18)	176 (20)	39,441	637
50–59 years	10,798 (32)	346 (27)	68,364	2089	10,511 (32)	287 (33)	67,281	1084
60–69 years	11,020 (32)	494 (39)	64,885	2901	10,759 (32)	261 (30)	63,951	934
70–79 years	4434 (13)	263 (21)	22,540	1208	4333 (13)	101 (12)	22,226	318
≥ 80 years	633 (2)	43 (3)	2296	144	621 (2)	12 (1)	2271	24
Menopausal status at diagnosis, *n* (%)								
Premenopausal	9378 (27)	≤ 195^2^ (−)	61,404	1158	9118 (27)	≤ 265^2^ (−)	60,452	952
Postmenopausal	24,774 (72)	1083 (85)	143,944	5937	24,176 (73)	598 (70)	141,816	2128
Unknown	39 (0)	≤ 5^2^ (−)	181	17	38 (0)	≤ 5^2^ (−)	180	2
Modified comorbidity status, *n* (%)^3^								
None	28,809 (84)	957 (75)	177,395	5510	28,093 (84)	716 (84)	174,781	2614
Low	4587 (13)	258 (20)	24,591	1356	4466 (13)	121 (14)	24,182	409
High	795 (2)	57 (4)	3542	245	773 (2)	22 (3)	3484	58
Tumour size, *n* (%)								
≤ 20 mm	20,756 (61)	≤ 775^2^ (−)	128,360	4479	20,248 (61)	≤ 515^2^ (−)	126,463	1896
21–50 mm	12,202 (36)	480 (38)	71,284	2497	11,873 (36)	329 (38)	70,186	1098
≥ 51 mm	1045 (3)	23 (2)	4878	113	1026 (3)	19 (2)	4795	83
Unknown	188 (1)	≤ 5^2^ (−)	1007	22	185 (1)	≤ 5^2^ (−)	1003	4
Lymph node status, *n* (%)								
N0	18,396 (54)	724 (57)	111,451	4040	17,986 (54)	410 (48)	109,988	1463
N1–3	10,391 (30)	353 (28)	66,956	2145	10,090 (30)	301 (35)	65,833	1123
N4+	5404 (16)	195 (15)	27,121	927	5256 (16)	148 (17)	26,625	496
UICC stage, *n* (%)								
I	13,433 (39)	519 (41)	81,013	2939	13,139 (39)	294 (34)	79,950	1063
II	15,067 (44)	551 (43)	95,812	3206	14,654 (44)	413 (48)	94,303	1509
III	5691 (17)	202 (16)	28,704	967	5539 (17)	152 (18)	28,194	510
Histological grade, *n* (%)^4^								
Low	9740 (28)	364 (29)	59,627	2029	9500 (29)	240 (28)	58,749	878
Moderate	12,742 (37)	507 (40)	78,631	2918	12,397 (37)	345 (40)	77,351	1280
High	6608 (19)	226 (18)	37,186	1170	6446 (19)	162 (19)	36,641	545
Unknown	5101 (15)	175 (14)	30,085	995	4989 (15)	112 (13)	29,705	379
ER status, *n* (%)								
ER negative (0–9%)	6376 (19)	224 (18)	35,446	1111	6198 (19)	178 (21)	34,822	624
ER positive (≥ 10%)	26,954 (79)	1031 (81)	164,836	5894	26,294 (79)	660 (77)	162,448	2388
Unknown	861 (3)	17 (1)	5247	106	840 (3)	21 (3)	5176	70
HER-2 status, *n* (%)^5^								
Negative	11,551 (34)	474 (37)	63,723	2370	11,245 (34)	306 (36)	62,732	992
Positive	2489 (7)	114 (9)	13,508	599	2426 (7)	63 (7)	13,317	191
Unknown	20,151 (59)	684 (54)	128,298	4143	19,661 (59)	490 (57)	126,398	1900
Endocrine therapy and ER status, *n* (%)								
ET−/ER−	7010 (21)	231 (18)	39,283	1154	6819 (20)	191 (22)	38,615	667
ET+/ER+	17,592 (51)	679 (53)	113,800	4123	17,109 (51)	483 (56)	112,074	1725
ET−/ER+	9362 (27)	352 (28)	51,037	1771	9185 (28)	177 (21)	50,374	663
ET+/ER−	227 (1)	10 (1)	1410	64	219 (1)	8 (1)	1384	26
Type of primary surgery, *n* (%)								
Mastectomy without radiotherapy	11,337 (33)	454 (36)	65,458	2379	11,101 (33)	236 (27)	64,585	873
Mastectomy with radiotherapy	6897 (20)	187 (15)	41,418	1045	6692 (20)	205 (24)	40,708	710
Lumpectomy with radiotherapy	15,957 (47)	631 (50)	98,653	3688	15,539 (47)	418 (49)	97,154	1499
Systemic therapy, *n* (%)								
No	2091 (6)	73 (6)	10,265	269	2046 (6)	45 (5)	10,089	176
Yes	32,100 (94)	1199 (94)	195,265	6843	31,286 (94)	814 (95)	192,358	2906
Chemotherapy, *n* (%)								
No	23,233 (68)	965 (76)	135,848	5277	22,713 (68)	520 (61)	133,933	1915
Yes	10,958 (32)	307 (24)	69,681	1835	10,619 (32)	339 (39)	68,514	1167
Endocrine therapy, *n* (%)								
No	16,117 (47)	577 (45)	88,710	2885	15,754 (47)	363 (42)	87,396	1314
Yes	18,074 (53)	695 (55)	116,819	4227	17,578 (53)	496 (58)	115,051	1768
Radiotherapy, *n* (%)								
No	11,337 (33)	454 (36)	65,458	2379	11,101 (33)	236 (27)	64,585	873
Yes	22,854 (67)	818 (64)	140,071	4733	22,231 (67)	623 (73)	137,862	2209
Co-medication at baseline, *n* (%)								
Simvastatin user	1791 (5)	135 (11)	9321	716	1753 (5)	38 (4)	9217	103
Aspirin user	447 (1)	23 (2)	2540	117	439 (1)	8 (1)	2503	36

^1^In person-years. ^2^According to Danish Data Protection Law, cell with very few individuals are not allowed to be presented. ^3^Charlson Comorbidity Index (CCI) without cancer included (low: score of 1 or 2; high: score of 3 or more). ^4^Histological grade is based on a composite score including tubule formation, mitoses, and nuclear pleomorphy, all consistent of a score of 1, 2, or 3. The scores are summarised, and a total score of 3–5 is categorised as low grade, 6–7 as moderate grade, and 8–9 as high grade (http://www.dbcg.dk/PDF%20Filer/Kap_3_Patologi_22_juni_2017.pdf). ^5^Systematic recording of HER-2 status started in 2007 HER-2 is classified according to immunohistochemistry (Hercept test) and by fluorescent in situ hybridisation (FISH) (counts 60 dots, yet min 6 cells and max 60 cells). The ratio is given as gene/chromosome with 2 decimals. HER-2 positive includes ‘HER-2 score = 3 and FISH ≤ 2.00’. HER-2 negative includes ‘HER-2 score of 0, 1, or 2 and FISH ≤ 2.00’

During follow-up, 859 (2%) of the 34,191 women with normal thyroid at baseline developed hypothyroidism in a median follow-up time of 3.4 years. Compared with women with normal thyroid function, women who developed hypothyroidism during follow-up had a higher frequency of lymph node involvement and were more likely to be assigned chemo-, radio-, and/or endocrine therapy.

### Recurrence

In total, 5626 (16%) women with normal thyroid function and 184 (14%) women with prevalent hypothyroidism developed recurrent breast cancer including 61% and 62% distant recurrences, respectively. Among women with incident hypothyroidism, 79 (9%) developed recurrence during follow-up of which 62% were distant recurrences.

After adjusting for potential confounding factors, women with prevalent hypothyroidism had a similar risk of recurrence as women with normal thyroid function (adjusted HR_prevalent_ 1.01, 95% CI 0.87–1.19). Likewise, there was little evidence of an association of incident hypothyroidism with breast cancer recurrence compared with normal thyroid function (adjusted HR_incident_ 0.93, 95% CI 0.75–1.16) (Table 2).

**Table 2:** Breast cancer recurrence and all-cause mortality, HR, and associated 95% CIs for women diagnosed with stage I–III, operable breast cancer in Denmark from 1996 to 2009 by hypothyroidism present at breast cancer diagnosis (prevalent) or during follow-up (incident)

	Recurrence					All-cause mortality				
	Women with breast cancer (counts (%))	Recurrent events (counts (%))	Follow-up time (years)	HR (95% CI)		Women with breast cancer (counts (%))	All-cause mortality (counts (%))	Follow-up time (years)	HR (95% CI)	
				Crude	Adjusted				Crude	Adjusted
Prevalent hypothyroidism										
Normal thyroid	34,191 (96)	5626 (16)	20,288	1.00	1.00	34,225 (96)	9696 (96)	36,907	1.00	1.00
Prevalent hypothyroidism	1272 (4)	184 (14)	594	0.94 (0.81–1.09)	1.01 (0.87–1.19)^1^	1273 (4)	398 (4)	1555	1.25 (1.13–1.39)	1.02 (0.92–1.14)^1^
Incident hypothyroidism										
Normal thyroid	33,332 (97)	5547 (17)	20,075	1.00	1.00	32,827 (96)	9422 (97)	36,599	1.00	1.00
Incident hypothyroidism	859 (3)	79 (9)	213	1.00 (0.80–1.24)	0.93 (0.75–1.16)^2^	1398 (4)	274 (3)	307	1.15 (1.02–1.30)	1.08 (0.95–1.23)^1^

^*HR* hazard ratio^

^1^Adjusted for age at diagnosis, UICC stage, ER/ET use, surgery type, receipt of chemotherapy, menopausal status, comorbidity, histologic grade, and use of simvastatin or aspirin (as time-varying covariates updated daily and lagged by 1 year), respectively. ^2^Adjusted for age at diagnosis, UICC stage, ER/ET status, type of primary surgery, and chemotherapy

The findings were similar in pre-planned sensitivity analysis with drug exposures lagged by 2 years (data not shown). Stratifying by menopausal status, ER status, and receipt of chemotherapy and radiotherapy produced little change to the effect estimates as shown in Table 3. Restricting the analysis to patients who had hypothyroidism 2, 5, and 10 years before breast cancer diagnosis did not alter the results substantially (adjusted HR_2year_ 1.00, 95% CI 0.84–1.18; adjusted HR_5year_ 0.88, 95% CI 0.71–1.10; adjusted HR_10year_ 0.91, 95% CI 0.63–1.31). The sensitivity analyses omitting surgery type and chemotherapy from the adjusted model did not alter the results (please see Additional file 2).

**Table 3:** Breast cancer recurrence and all-cause mortality, HR, and 95% CIs associating hypothyroidism status among stage I–III, operable breast cancer women diagnosed from 1996 to 2009 stratified by menopausal status, ER status, and chemo- and radiotherapy

	Prevalent hypothyroidism			Incident hypothyroidism		
**Recurrence**	**Recurrence (counts)**	**HR (95% CI)**		**Recurrence (counts)**	**HR (95% CI)**	
		Crude	Adjusted^1^		Crude	Adjusted^2^
Menopausal status						
Premenopausal	1653	1.00 (0.70–1.43)	1.15 (0.79–1.69)	1622	1.30 (0.91–1.87)	1.24 (0.86–1.78)
Postmenopausal	4152	0.93 (0.79–1.09)	0.99 (0.83–1.17)	3999	0.87 (0.66–1.16)	0.82 (0.61–1.08)
ER status						
Positive	4236	0.94 (0.79–1.11)	1.00 (0.83–1.19)	4099	0.97 (0.75–1.25)	0.93 (0.71–1.20)
Negative	1391	1.04 (0.77–1.39)	1.05 (0.76–1.45)	1346	1.08 (0.68–1.73)	1.02 (0.64–1.62)
Chemotherapy^3^						
Yes	–	–	–	1950	1.01 (0.72–1.50)	0.97 (0.67–1.40)
No	–	–	–	3676	0.98 (0.74–1.29)	0.91 (0.69–1.21)
Radiotherapy^3^						
Yes	–	–	–	3643	0.96 (0.73–1.25)	0.91 (0.69–1.20)
No	–	–	–	1983	1.11 (0.75–1.63)	0.99 (0.67–1.46)
**All-cause mortality**	**Mortality (counts)**	**HR (95% CI)**		**Mortality (counts)**	**HR (95% CI)**	
		Crude	Adjusted^1^		Crude	Adjusted^1^
Menopausal status						
Premenopausal	1792	1.03 (0.73–1.47)	1.19 (0.82–1.71)	1760	1.24 (0.94–1.64)	1.15 (0.85–1.55)
Postmenopausal	8298	1.17 (1.05–1.30)	1.00 (0.89–1.12)	7932	1.13 (0.99–1.29)	1.06 (0.91–1.23)
ER status						
Positive	7418	1.25 (1.11–1.40)	1.00 (0.88–1.13)	7118	1.13 (0.99–1.30)	1.06 (0.91–1.23)
Negative	2354	1.32 (1.07–1.63)	1.20 (0.95–1.50)	2263	1.27 (0.98–1.64)	1.24 (0.93–1.64)
Chemotherapy						
Yes	2486	1.17 (0.92–1.48)	1.15 (0.90–1.48)	2416	1.18 (0.91–1.51)	1.12 (0.85–1.47)
No	7608	1.23 (1.11–1.38)	0.98 (0.87–1.11)	7280	1.16 (1.00–1.33)	1.06 (0.91–1.23)
Radiotherapy^3^						
Yes	–	–	–	5316	1.13 (0.96–1.33)	1.08 (0.90–1.29)
No	–	–	–	4380	1.22 (1.02–1.46)	1.11 (0.91–1.34)

*HR* hazard ratio

^1^Adjusted for age at diagnosis, menopausal status, UICC stage, histologic grade, ER/ET use, surgery type, receipt of radio- and chemotherapy, comorbidity, and use of simvastatin or aspirin, respectively. ^2^Adjusted for age at diagnosis, UICC stage, ER/ET use, surgery type, and receipt of chemotherapy. ^3^Only evaluated for incident hypothyroidism

### All-cause mortality

The study cohort for all-cause mortality included a further 35 women with breast cancer that had been excluded previously due to incomplete follow-up for recurrence. Overall, 10,094 women with breast cancer died during the study period of whom 398 (3%) were women with prevalent hypothyroidism and 274 (3%) were women who developed hypothyroidism during follow-up.

Women with prevalent hypothyroidism had a higher mortality risk than women with normal thyroid function (crude HR_prevalent_ 1.25, 95% CI 1.13–1.39), but the association attenuated after adjusting for confounders (adjusted HR_prevalent_ 1.02, 95% CI 0.92–1.14)—histological grade, UICC stage, type of surgery, and comorbidity burden.

Compared with women with normal thyroid function, women with incident hypothyroidism had a slightly increased risk of dying (crude HR_incident_ 1.15, 95% CI 1.02–1.30), which attenuated after adjusting for confounders (adjusted HR_incident_ 1.08, 95% CI 0.95–1.23)—histological grade, UICC stage, type of surgery, and comorbidity burden.

Stratifying by menopausal status, ER status, and receipt of chemotherapy and radiotherapy did not alter these findings (Table 3). Furthermore, restricting to patients with prevalent hypothyroidism in 2, 5, and 10 years, or drug exposures lagged by 2 years did not affect the estimates substantially (data not shown).

## Discussion

Evidence from our large cohort study does not support an association between hypothyroidism present at the time of diagnosis or during follow-up and breast cancer recurrence and all-cause mortality. For both recurrence and all-cause mortality, the near-null findings were not modified after stratification by menopausal status, ER status, chemotherapy, or radiotherapy or by duration of hypothyroidism prior to breast cancer diagnosis.

Our study has several strengths. We studied a large, nationwide cohort of women with breast cancer treated in a tax-supported and uniformly organised health care system with complete follow-up. Thus, selection bias seems unlikely. Furthermore, all breast cancer patients registered in the DBCG undergo standardised medical follow-up visits up to 10 years after primary diagnosis and any recurrent breast cancers are systematically registered in the DBCG [24]. Overall, 77% of patients diagnosed with an incident breast cancer from 2006 to 2015 attended the entire follow-up programme with higher attendance among younger patients (~ 81%) compared with patients aged over 75 years (~ 74%). In addition, the systematic collection of data on clinical, tumour, and treatment characteristics on all breast cancer patients enabled us to account for important potential confounders that could affect the risk of recurrence and mortality. The completeness of the DNPR is high [28]. The positive predictive value is 80, in general, and higher for conditions that always lead to hospitalisation. However, for conditions like hypothyroidism, the completeness may not be as high as this is often treated outside a hospital setting by a general practitioner. We therefore supplemented our study using data from the Danish National Prescription Registry. In the models of incident hypothyroidism, we used a time-varying approach to eliminate immortal time bias and lagged the exposure to eliminate reverse causation [31, 35].

Our study is also subject to some limitations. Hypothyroidism is underreported in the general population probably due to non-specific symptoms such as weight gain, fatigue, and memory loss, all of which may increase with age [4, 36]. In this study, we defined hypothyroidism from diagnosis codes or redeemed prescriptions but not by measures of hormone levels in blood samples as these were unavailable. Therefore, we cannot comment on the relationship between underlying hormone levels and breast cancer recurrence. In addition, we had no information on untreated subclinical hypothyroidism. We therefore cannot rule out the likelihood of undiagnosed and thus misclassified hypothyroidism among the women with breast cancer, which may bias our findings towards the null. Furthermore, our findings may be prone to residual confounding—for example, information on medication use (dosage and prescription compliance) and lifestyle factors associated with breast cancer or hypothyroidism (smoking, obesity, and physical activity) were not captured by the registries [37, 38]. Given the absence of data on actual hormone levels, an analysis comparing actual thyroid hormone values to recurrence risk could not be performed. Last, our definition of hypothyroidism was based on diagnostic codes and/or prescriptions for levothyroxine substitution therapy. As such, the impact of treating hypothyroidism may dilute the effect of hypothyroidism on recurrence. In total, 96.7% of the women with hypothyroidism in our study were on levothyroxine substitution. This may be a potential reason why our hypothesis of a positive effect of hypothyroidism on recurrence, as suggested by the laboratory models, was not confirmed [20, 21].

Previous studies on the association of thyroid function with survival in breast cancer patients have compared survival according to levels of thyroid hormones [39, 40] or using cancer-free controls [37, 41, 42]. To our knowledge, only two studies have investigated the association of hypothyroid disease with survival in a cohort of breast cancer patients [43, 44]. However, one of these—the Malmø Diet and Cancer Study by Brandt et al.—is not comparable to ours as thyroid hormone measurements were collected at the time of inclusion into the study, on average 5 years before the time of breast cancer diagnosis [44]. The study by Fiore et al. only included breast cancer patients with aggressive tumours and performed blood tests to assess thyroid function after surgery and before treatment [43]. By measuring actual levels of thyroid hormone, they were able to detect not only overt hypothyroidism but also subclinical hypothyroidism. However, their study was small, including only 47 patients and only two cases of subclinical hypothyroidism; thus, estimates were presented for all types of thyroid dysfunction. Similarly, a study by Jiskra et al. was hampered by a small sample size (84 patients) and consequently had a low number of cases with hypothyroidism [37]. Jiskra et al. also found no association of levels of thyroid hormones with relapse-free or overall survival in breast cancer patients compared with cancer-free controls. However, these latter two studies were likely underpowered for the hypothyroidism-breast cancer association. Thus, our prospective cohort study is the first to distinguish between the association of prevalent and incident hypothyroidism on the risk of breast cancer recurrence and overall mortality.

In our data, we note that 91% of patients with hypothyroidism were retrieved from the prescription registry, while the remainder were ascertained based on hospital diagnoses of hypothyroidism. This is not surprising as most cases of hypothyroidism are likely to be diagnosed and treated by a general practitioner. Furthermore, this highlights the importance of considering both diagnostic codes and prescription medications in future studies on hypothyroidism.

## Conclusion

This prospective cohort study suggests that hypothyroidism present at the time of diagnosis or incident during follow-up is not associated with breast cancer recurrence or all-cause mortality. From a clinical point of view, this is reassuring for patients who suffer from hypothyroidism and for their physicians highlighting that hypothyroidism is unlikely to have an unfavourable impact on the clinical course of breast cancer or survival.

## Additional files

Additional file 1: Time lagging exposure. (PDF 164 kb)

Additional file 2: Sensitivity analyses. (PDF 124 kb)

Additional file 3: DAG for incident hypothyroidism. (PDF 336 kb)

## Abbreviations

ATC: Anatomical Therapeutic Classification
CI: Confidence interval
DBCG: Danish Breast Cancer Group
DNPR: Danish National Prescription Registry
DNRP: Danish National Registry of Patients
ER: Oestrogen receptor
ET: Endocrine therapy
HR: Hazard ratio
ICD: International Classification of Diseases
