# Supplementary material for: Hypothyroidism and the risk of breast cancer recurrence and all-cause mortality - a Danish population-based study
Source: Breast Cancer Res. 2019 Mar 22;21:44. doi: 10.1186/s13058-019-1122-3 (PMC6431068; doi:10.1186/s13058-019-1122-3)
Supplement: Supplementary file 1 — Time lagging exposure. (PDF 164 kb) [file 13058_2019_1122_MOESM1_ESM.pdf]

**ADDITIONAL FILE 1: Time lagging exposure**

In the Figure below, we illustrate the person-time lag and how this operationalized in the incident model. The **pink line** represents person-time for women with no redeemed prescriptions and/or no hospital diagnosis code for hypothyroidism. This person-time is deemed unexposed and accordingly allocated "normal thyroid function". The **green line** and **yellow line** represent women with a hospital diagnosis code for hypothyroidism or redeemed levothyroxine prescription at some time during the study period. The **green line** starts at the date of hospital code or second redeemed prescription and lasts for one year. During this first year after hypothyroidism diagnosis code or prescription, the person-time is allocated to the "normal thyroid function" (therefore unexposed) to minimize reverse causation. The one-year lag means that after the end of this first year, the patient with a hypothyroidism diagnosis code or prescription then starts to contribute exposed person-time in the models. This method is recommended in pharmacoepidemiological studies to avoid immortal person-time bias [1].

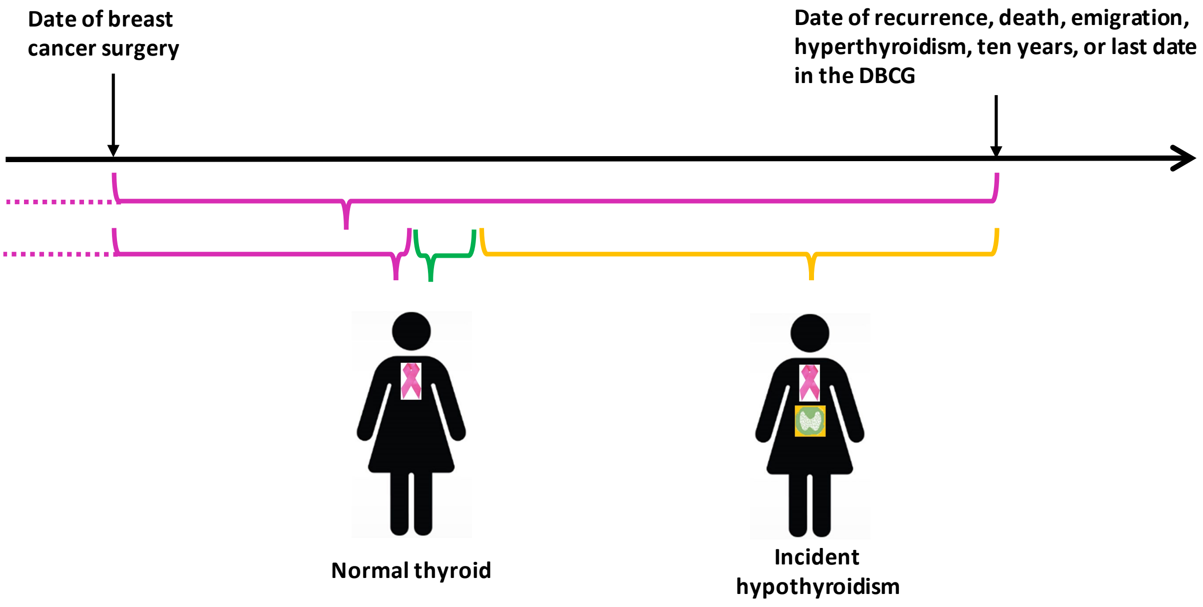


**References:**

1. Suissa S: **Immortal time bias in pharmaco-epidemiology**. *American journal of epidemiology* 2008, **167**(4):492-499.
