# Supplementary material for: Hypothyroidism and the risk of breast cancer recurrence and all-cause mortality - a Danish population-based study
Source: Breast Cancer Res. 2019 Mar 22;21:44. doi: 10.1186/s13058-019-1122-3 (PMC6431068; doi:10.1186/s13058-019-1122-3)
Supplement: Supplementary file 2 — Sensitivity analyses. (PDF 124 kb) [file 13058_2019_1122_MOESM2_ESM.pdf]

ADDITIONAL FILE 2: Sensitivity analyses

In the sensitivity analyses, we performed the main analysis without adjusting for any of the treatment modalities chemotherapy, radiotherapy and surgery. As seen in the Table, these new results show similar results to the main analyses.

	RECURRENCE – presented in the paper					Without adjustment for treatment modalities
	Women with breast cancer	Recurrent events	Follow-up time	HR (95% CI)		
	Counts (%)	Counts (%)	(Years)	Crude	Adjusted	Adjusted
**PREVALENT MODEL**						
Normal thyroid	34 191 (96)	5 626 (97)	20 288	1.00	1.00	1.00
Prevalent hypothyroidism	1 272 (4)	184 (3)	594	0.94 (0.81-1.09)	1.01 (0.87-1.19)	1.00 (0.85-1.16)
**INCIDENT MODEL**						
Normal thyroid	33 332 (97)	5 547 (99)	20 075	1.00	1.00	1.00
Incident hypothyroidism	859 (3)	79 (1)	213	1.00 (0.80-1.24)	0.93 (0.75-1.16)	0.92 (0.74-1.15)
	**ALL-CAUSE MORTALITY – presented in the paper**					**Without treatment modalities**
**PREVALENT MODEL**						
Normal thyroid	34 225 (96)	9 696 (96)	36 907	1.00	1.00	1.00
Incident hypothyroidism	1 273 (4)	398 (4)	1 555	1.25 (1.13-1.39)	1.02 (0.92-1.14)	1.01 (0.90-1.12)
**INCIDENT MODEL**						
Normal thyroid	32 827 (96)	9 422 (97)	36 599	1.00	1.00	1.00
Incident hypothyroidism	1 398 (4)	274 (3)	307	1.15 (1.02-1.30)	1.08 (0.95-1.23)	1.08 (0.94-1.22)
