# Supplementary material for: Hypothyroidism and the risk of breast cancer recurrence and all-cause mortality - a Danish population-based study
Source: Breast Cancer Res. 2019 Mar 22;21:44. doi: 10.1186/s13058-019-1122-3 (PMC6431068; doi:10.1186/s13058-019-1122-3)
Supplement: Supplementary file 3 — DAG for incident hypothyroidism. (PDF 336 kb) [file 13058_2019_1122_MOESM3_ESM.pdf]

ADDITIONAL FILE 3: DAG for incident hypothyroidism

Figure 1: Directed acyclic graph (DAG) used to identify the relevant covariates in the incident model due to the low number of recurrent events among breast cancer women with incident hypothyroidism.

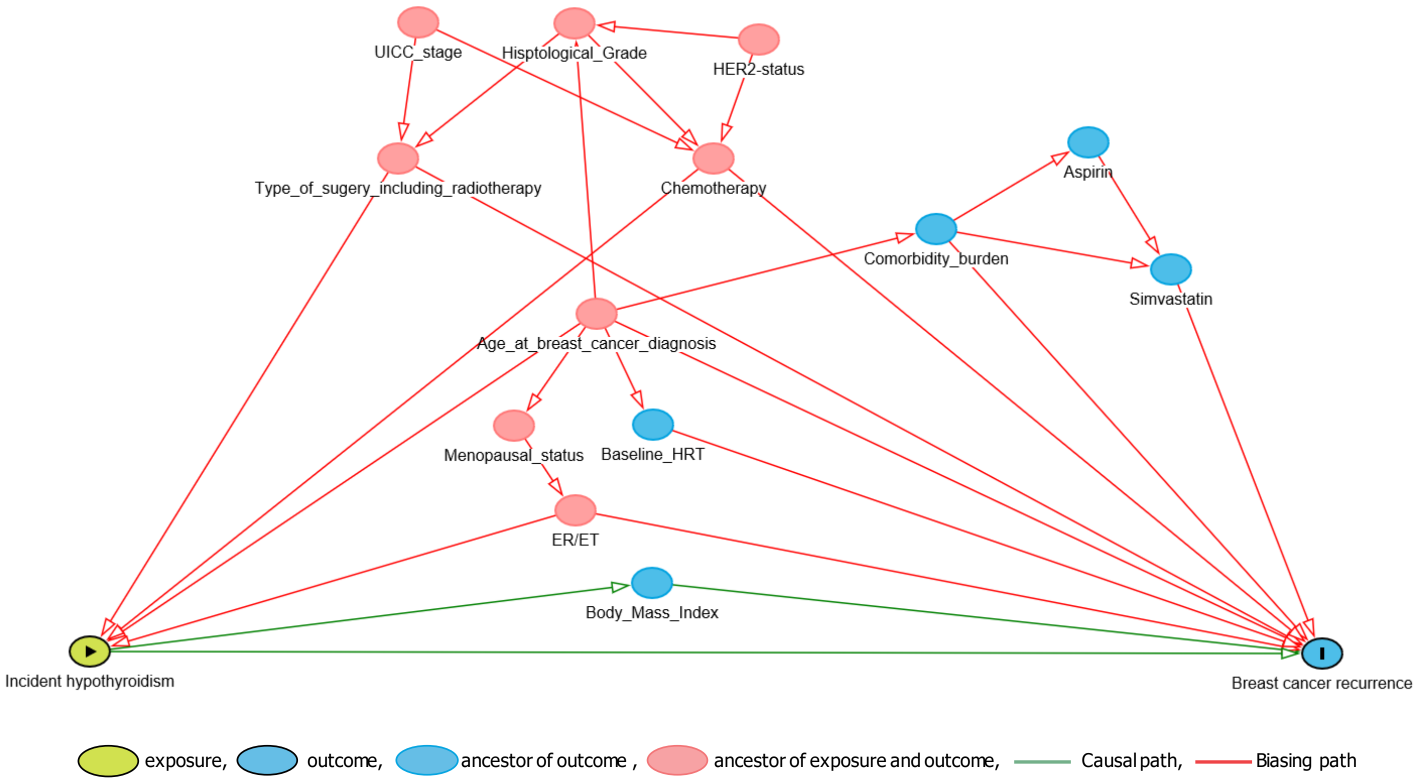

Based on the DAG, the minimal sufficient adjustment sets for estimating the total effect of incident hypothyroidism on breast cancer recurrence included the five covariates listed below. Their associations with incident hypothyroidism and breast cancer recurrence were based on following hypotheses:

<u>Age at breast cancer diagnosis:</u>

- Older patients have an increased risk of developing hypothyroidism [1].
- Younger breast cancer patients are at higher risk of recurrence [2].

<u>UICC stage:</u>

- Patients with more advanced UICC stage have an increased risk of developing hypothyroidism particularly through treatment modalities as they are likely to receive more aggressive therapy.
- Patients with more advanced UICC stage are at higher risk of recurrence [3].

<u>Type of primary surgery:</u>

- Patients treated with surgery including radiotherapy have an increased risk of developing hypothyroidism [4-6].

- Patients treated with surgery including radiotherapy are at higher risk of recurrence due to more advanced breast cancer.

Chemotherapy:

- Patients treated with chemotherapy have an increased risk of developing hypothyroidism [7-10].
- Patients treated with chemotherapy are at higher risk of recurrence due to more advanced breast cancer.

ER/ET status:

- Patients with oestrogen receptor positive breast cancer treated with endocrine therapy are at increased risk of developing hypothyroidism.
- Patients with oestrogen receptor positive breast cancer treated endocrine therapy are at lower risk of recurrence because they receive the treatment appropriate for this type of cancer.

REFERENCES:

1. Garmendia Madariaga A, Santos Palacios S, Guillen-Grima F, Galofre JC: **The incidence and prevalence of thyroid dysfunction in Europe: a meta-analysis**. *The Journal of clinical endocrinology and metabolism* 2014, **99**(3):923-931.
2. Mahmood H, Faheem M, Mehmood S: **Association of menopausal status with pathological features of tumor in stage I to IIIA breast cancer patients treated with upfront modified radical mastectomy**. *J Cancer Prev Curr Res* 2015, **4**(1):000109.
3. McBain CA, Young EA, Swindell R, Magee B, Stewart AL: **Local recurrence of breast cancer following surgery and radiotherapy: incidence and outcome**. *Clinical oncology (Royal College of Radiologists (Great Britain))* 2003, **15**(1):25-31.
4. Reinertsen KV, Cvancarova M, Wist E, Bjoro T, Dahl AA, Danielsen T, Fossa SD: **Thyroid function in women after multimodal treatment for breast cancer stage II/III: comparison with controls from a population sample**. *International journal of radiation oncology, biology, physics* 2009, **75**(3):764-770.
5. Smith GL, Smith BD, Giordano SH, Shih YC, Woodward WA, Strom EA, Perkins GH, Tereffe W, Yu TK, Buchholz TA: **Risk of hypothyroidism in older breast cancer patients treated with radiation**. *Cancer* 2008, **112**(6):1371-1379.
6. Bruning PF, Bonfrer JG, Engelsman E, Hamersma-vd Linden E, de Jong-Bakker M, Nooyen W: **Pros and cons of aminoglutethimide for advanced postmenopausal breast cancer**. *Breast cancer research and treatment* 1984, **4**(4):289-295.
7. Duarte R, De Luis DA, Lopez-Jimenez J, Roy G, Garcia A: **Thyroid function and autoimmunity during treatment with G-CSF**. *Clinical endocrinology* 1999, **51**(1):133-134.
8. Fentiman IS, Balkwill FR, Thomas BS, Russell MJ, Todd I, Bottazzo GF: **An autoimmune aetiology for hypothyroidism following interferon therapy for breast cancer**. *European journal of cancer & clinical oncology* 1988, **24**(8):1299-1303.
9. de Groot S, Janssen LG, Charehbili A, Dijkgraaf EM, Smit VT, Kessels LW, van Bochove A, van Laarhoven HW, Meershoek-Klein Kranenbarg E, van Leeuwen-Stok AE *et al*: **Thyroid function alters during neoadjuvant chemotherapy in breast cancer patients: results from the NEOZOTAC trial (BOOG 2010-01)**. *Breast cancer research and treatment* 2015, **149**(2):461-466.
10. Mamby CC, Love RR, Lee KE: **Thyroid function test changes with adjuvant tamoxifen therapy in postmenopausal women with breast cancer**. *Journal of clinical oncology : official journal of the American Society of Clinical Oncology* 1995, **13**(4):854-857.
